# Spiking network modeling of neuronal dynamics in individual rats

**DOI:** 10.1186/1471-2202-16-S1-P122

**Published:** 2015-12-18

**Authors:** John S Choi, Rosemary J Menzies, Salvador Dura-Bernal, Joseph T Francis, William W Lytton, Cliff C Kerr

**Affiliations:** 1 Department of Physiology and Pharmacology, SUNY Downstate Medical Center, Brooklyn, NY 10029, USA; 2 Complex Systems Group, School of Physics, University of Sydney, Sydney, NSW 2006, Australia; 3 Centre of Excellence for Integrative Brain Function, University of Sydney, Sydney, NSW 2006, Australia

## 

Many researchers base their neuronal models on experimental data, but few systematically calibrate to it, and even fewer use unpooled data from multiple subjects. Some (partial) exceptions are [1], where one model parameter was calibrated to data pooled across four macaques; and [2], where five model parameters were calibrated to unpooled data from 292 humans. To our knowledge, no one has previously calibrated spiking network models to individual subjects - likely because a single model iteration typically takes considerable computational time, compounded by the formidable number of iterations required to perform optimizations on a statistically meaningful number of subjects.

In this study, we calibrate and validate a mesoscopic spiking network model against data from individual rats, then use these fits to infer differences in the rats' physiologies. We recorded data from microelectrode arrays implanted in the somatosensory cortices of nine male rats, each of whom received touch stimuli to his left forepaw. The spiking network models consisted of 20,000 Izhikevich neurons, representing a 2x2 mm patch of cortex sampled at approximately 10% true cell density, with cell types (15 across six layers) and connectivities based on empirical data. We calibrated key model parameters (including connection probability and weight, tonic background activity, and the ratio of thalamic to cortical input) to experimental data (including average firing rates and coefficients of variation) using a nonlinear optimization algorithm [3]. The calibrations were validated using the exponents of the local field potential (LFP) power spectra from 5-50 Hz, which were not used for calibration. Changes in the information processing properties of the (simulated) networks were then quantified via interlaminar Granger causality.

Experimentally, we found large differences between subjects. Cortical firing rates varied from 3.8 Hz to 19.6 Hz (median 11.9 Hz); coefficients of variation, 0.4 to 1.2 (median 0.9); and peristimulus time histogram peak amplitudes, 48 Hz to 73 Hz (median 62 Hz). Inter-subject differences were significantly greater than intra-subject differences across both sessions and electrodes. The calibrated models reproduced experimental data with an average mismatch of 16%, compared to 30% when uncalibrated. Experimentally, the LFP power spectra exponents varied from -1.4 to -2.7 (median -2.2). While the models had uniformly steeper exponents (range -2.8 to -3.6), calibration reduced mismatch in eight of the nine subjects. Inter-subject differences could be largely accounted for by differences in average synaptic connection probability (which varied by a mean of 24% between subjects, compared to <8% for other fitted parameters). Parameter differences produced changes in how these networks process information - for example, there were significant and complex differences in the patterns of interlaminar information flow between subjects, including reversals in the dominant direction of information flow for some layer pairs.

In summary, we found that (1) significant differences exist between individual subjects, (2) these differences can be captured by calibrating spiking network models to data from each individual, and (3) modeling suggests that these differences affect how individuals process information across cortical layers.
